# Drug hypersensitivity to various antihistamines with cross-reactions: a case report

**DOI:** 10.1186/2045-7022-4-S3-P132

**Published:** 2014-07-18

**Authors:** Jaechun Lee, Young Uck Kim, Su Hee Kim

**Affiliations:** 1 Jeju National University School of Medicine, Korea, Republic of; 2 Jeju National University School of Medicine, Internal Medicine, Korea, Republic of

## 

Antihistamines (Histamine receptor antagonists) are widely prescribed medicines in the treatment of allergic disorders, especially the symptoms of hypersensitivity reactions, mainly blocking the activity of vasoactive amines to their receptors. Drug adverse reactions such as sleepiness and dry mouth are frequently encountered. However, drug hypersensitivity provoking itchy hives by antihistamines were rarely reported. A 41-year-old female patient visited allergy clinic for generalized itchy hives from time to time, which had been aggravated 3 months before. Whenever she was exposed to antihistamines for treatment, she felt her hives got immediately full-blown. As a screening, she was tested with various antihistamines on her skin, then skin test-negative antihistamines were orally administered. Finally we failed to choose a safe antihistamine for the treatment of her symptoms. We report a case of drug hypersensitivity to various antihistamines with cross-reactions in a patient with chronic urticaria.

